# Death under Anæsthetics

**Published:** 1889-12

**Authors:** 


### SELECTIONS.

### Deaths Under Anæsthetics.

The death of Lady Milne at Edinburgh, has given rise to a great deal of comment and unjust criticism of nitrous-oxide as an anæsthetic. There is a very great difference between a death occurring under an anæsthetic, and a fatality following directly as the result of the anæsthetic, and in the Edinburgh case this important difference is brought out into very bold relief. A lady, aged 71—described as looking somewhat unhealthy and flabby, is oppressed by nervous apprehension, and even goes so far as to tell her daughter that the visit to the dentist will be the death of her—comes to have gas administered dressed in such tight fitting clothing that she can not breathe without difficulty, and to add to these she has a stomach distended with undigested food; under these circumstances the wonder would rather be if death had not ensued than that Lady Milne died. With fatty degeneration of the heart syncope is of course an always possible occurrence, and the fact that a person is unable to breathe with normal fulness, and that the thoracic movements are in any way hampered must greatly enhance the danger of heart failure. We regret that the daily press should have got hold of the case in point; firstly, because its utterances on scientific matters are almost invariably inaccurate, unreliable and only too often inspired by personal favor or gain-getting; and secondly, because in these days of hurry and superficiality men are only too apt to accept with unquestioning credulity the statements made by their matutinal penny oracle. In this instance no exception has occurred, and the nonprofessional papers have mostly indulged in platitudes and alarmist paragraphs only too calculated to shake the confidence of the public in a useful anæsthetic, if not to promote doubtful substitutes. Apropos to these latter, we have received an amusing notification, that we are at liberty to publish, what a correspondent calls "a Yankee notion" for the promotion of anæsthesia by "vitalized air." Our correspondent's name and address are written large, but lest we should hurt his modesty by inducing crowds of aspirants after "vitalized air" anæsthesia, we refrain from giving them publicity; we might add for his private ear that "vitalized air" has been exploited a long time ago, and has even been exploded, and the charlatan who thought to rob the public by giving them an old remedy under a new name, has met with the fate which such gentry most richly deserve. Anæsthetics are many, but safe ones are few. Further, even the safest may be converted into the most lethal by carelessness and lack of precaution upon the part of the administrator, or even the patient. It is a great pity that the public is so ill-informed concerning anæsthetics, but probably it arises from the fact that medical men, from whom they derive most of their facts of a medical character and opinions, are themselves poorly versed in the history, physiological action, and methods of administration of anæsthetics.—*British Journal of Dental Science.*

